# Comparative efficacy of laser and electroacupuncture on anxiety management and salivary alpha-amylase levels in pediatric dental patients with excessive gag reflexes: a randomized clinical trial

**DOI:** 10.1186/s12903-025-06630-x

**Published:** 2025-07-26

**Authors:** Marwa Baraka, Sarah I. Zeitoun, Sara Salah, Sawsan Hafez

**Affiliations:** Faculty of Dentistry, Pediatric Dentistry and Dental Public Health Department, Alexandria University, Champollion St., El Azareta, Alexandria, Egypt

**Keywords:** Alginate impression, Gag reflex, Dental anxiety, Pediatric dentistry, Salivary amylase, Acupuncture, Laser

## Abstract

**Background:**

This study examines the effects of laser acupuncture and electroacupuncture on anxiety in children with excessive gag reflexes during dental impressions.

**Methods:**

A three-armed randomized controlled trial involved 63 pediatric dental patients aged 6–9 with excessive gag reflexes. Participants were equally divided into three groups: group 1 (laser acupuncture), group 2 (electroacupuncture), and group 3 (negative control with a deactivated device). Salivary alpha-amylase (sAA) levels, Frankl scores, facial image scale (FIS), and heart rate were measured before and after interventions to assess dental anxiety. Group comparisons were conducted using one-way ANOVA or Kruskal-Wallis tests, followed by Bonferroni-corrected pairwise comparisons for significant results, with significance defined as *p* < 0.05.

**Results:**

The study found no significant differences in sAA levels before the intervention, but electroacupuncture significantly reduced levels post-intervention (*p* = 0.009) compared to the laser and control groups. Frankl scores also improved significantly with electroacupuncture (*p* = 0.002), indicating better cooperation during dental procedures. Both electroacupuncture and laser groups reduced heart rates significantly compared to control (*p* < 0.001), with correlations indicating that higher sAA levels were associated with increased discomfort in the laser group, and that stress levels were linked to heart rate in the control group.

**Conclusions:**

Both laser acupuncture and electroacupuncture effectively lowered anxiety level in children with gag reflex. While both methods improved objective and subjective scores, electroacupuncture demonstrated superior efficacy in managing anxiety.

**Clinical relevance:**

These findings support acupuncture techniques as valuable tools in pediatric dentistry for improving patient cooperation and managing anxiety during dental procedures.

**Trial registration:**

Registered on clinicaltrial.gov; NCT06422286, 16/4/2024.

**Supplementary Information:**

The online version contains supplementary material available at 10.1186/s12903-025-06630-x.

## Background

Taking dental impressions is essential in pediatric dentistry, but patient compliance often poses challenges. Compliance is the key for effective communication, reducing anxiety, ensuring quality care, building trust between the dentist and parent, and fostering a positive attitude toward oral health. However, children with strong gag reflexes may experience hyperactive gagging episodes when taking alginate impressions of the upper arch [1]. 

The gag reflex (GR) can be triggered somatically (physically) by touching specific areas, serving as an involuntary mechanism to prevent foreign objects from entering the throat, controlled by nerve endings in the soft palate, throat, and posterior tongue [2, 3]. It involves the glossopharyngeal (CN IX) and vagus (CN X) cranial nerves, which exit the medulla via the jugular foramen to innervate the pharynx and soft palate [4]. The trigeminal nerve (CN V) can also trigger the reflex through soft palate stimulation. Additionally, GR can be triggered psychogenically by mental stimuli, such as being in a dental office, sensing dental-related aromas, or seeing a tongue depressor [3]. Other factors, including nasal obstruction, gastrointestinal disorders, and anatomical variations in the soft palate, may also contribute to an exaggerated reflex [5]. Excessive gagging during dental procedures often indicates resistance to treatment, linked to stress and anxiety from past negative experiences, suggesting a connection between gagging and dental phobia [6–8]. 

While gagging is a natural response, excessive GR can disrupt preliminary dental procedures, such as impressions [9]. This heightened response is time-consuming and can compromise treatment, leading to postponed procedures and increased stress for both dentist and patient. It may also result in future avoidance of dental care due to anxiety. Various methods have been suggested to manage GR, including relaxation, distraction, desensitization, behavioral therapies, local anesthesia, conscious sedation, general anesthesia, and complementary approaches like hypnosis, acupuncture, and acupressure [1, 3, 10]. 

Acupuncture, which involves inserting needles into specific body points for disease prevention and health maintenance, has been shown to boost serotonin production in the brain, potentially helping to regulate the GR by sending impulses to the midbrain’s control centers, particularly the Nucleus Raphe Magnus, a key source of serotonin (5-HT) which is then metabolized to beta-endorphins with an antiemetic function [11]. Pericardium 6 (PC6) is one of the best-introduced acupressure locations for nausea and vomiting management. It is a concave region on the medial part of the forearm, almost below the palm, with a width of about one horizontal finger [9, 12]. 

PC6 can be stimulated via needles, pressure (acupressure), vacuum, laser, or electrical stimulus (electro-acupuncture) [13]. Due to the invasiveness of needle acupuncture, it’s challenging for children, making laser acupuncture and electroacupuncture effective, pain-free alternatives for managing exaggerated GR, reducing anxiety, and improving compliance. Laser acupuncture (622 to 1322 nm) operates at low output power, ranging between 1 and 100 milliwatts (mW), allowing light to penetrate tissue and activate cellular chromophores, generating an electrochemical potential that boosts ATP synthesis and enhances RNA, DNA, and protein production, known as Photobiomodulation [11]. Similarly, microcurrent electrical stimulation applies low-frequency electrical stimulation to skin receptors at specific acupoints, modulating the GR like needle acupuncture [14]. 

Biomarkers in oral fluids provide objective insights into various bodily processes and have important diagnostic and prognostic implications, facilitating the development of “oral-based diagnostics” [15]. Key systems involved in the body’s stress response include the hypothalamic-pituitary-adrenal (HPA) axis and the sympathetic nervous system [16]. Two primary saliva biomarkers, effective stress indicators, are salivary cortisol, associated with the HPA axis, and salivary α-amylase (sAA), linked to the sympathetic nervous system [17–21]. 

Current studies show insufficient evidence regarding the efficacy of laser acupuncture and electroacupuncture in managing anxiety levels in children with moderate to severe gag reflexes, and whether one method is superior. This study aims to evaluate and compare the effects of low-power laser therapy and microcurrent electrical stimulation on anxiety by measuring sAA levels in children with excessive gag reflexes during dental impressions. Additionally, it seeks to correlate changes in sAA levels with subjective anxiety measures, such as the facial image scale and Frankl rating scale, as well as objective measures like heart rate. The null hypothesis posits no significant difference between the two methods in controlling anxiety during dental impressions.

## Methods

### Study design

This three-armed randomized controlled trial (1:1:1 allocation) followed IRB guidelines at the Faculty of Dentistry, Alexandria University (IRB NO 00010556- IORG 0008839) and was registered on clinicaltrial.gov (NCT06422286), adhering to CONSORT guidelines [22]. The PICO question was: In children aged 6–9 with moderate to severe gag reflex (Population), do laser acupuncture (Intervention I) or electroacupuncture (Intervention II) reduce sAA levels compared to a negative control group (Control)? Recruitment involved written informed consent from the legal guardians of all participants to participate in the study.

### Study setting

Participants were recruited from patients attending the Pediatric Dentistry Clinic, Faculty of Dentistry, Alexandria University. The study was carried out at the Laser Dental Clinics of the same university.

### Sample size

Sample size was calculated with a 5% alpha error and 80% power. Sari et al. [3] reported mean (SD) Gagging Preventive Index (GPI) during impression taking as 2.75 ± 1.75 for the laser group, 1.75 ± 0.85 for the acupressure group, and 3.50 ± 1.10 for the control group. Salivary amylase secretion may increase during GR [23]. Using the highest SD for adequate power, the minimum sample size was determined to be 21 patients per group, totaling 63 patients (3 groups × 21) [24]. Software used: G*Power (Version 3.1.9.7).

### Eligibility criteria

Children aged 6–9 years, without systemic diseases or special health care needs [10], and scoring 2 or 3 on the Frankl Behavioral Rating Scale [25], were selected for the study. They required a full arch maxillary alginate impression and had gagging severity index (GSI) grades III to V (Supplementary Table 1), assessed with a tongue blade [1, 5]. Children taking anti-emetic drugs or sensitive to alginate and those who have any neurodevelopmental disorders (e.g., autism spectrum disorder, intellectual disability) were excluded [11]. 

### Randomization, allocation concealment and blindness

Randomization was conducted using a computer-generated allocation software (Sealed Envelope). Each child received a serial number linked to their assigned group, written on identical sheets and placed in opaque envelopes. A trial-independent personnel managed the envelopes, revealing them only during the dental examination to keep the operator unaware of group assignments. Both the participants and the statistician were blinded to the gagging control technique, though the operator could not be blinded.

### Grouping

The children fulfilling the inclusion criteria were randomly divided into 3 groups according to the method that was used to control GR:

- **Group I (Study group)**: the children were allocated to low-power laser (*N* = 21).
- **Group II (Study group)**: the children were allocated to microcurrent electrical stimulation (*N* = 21).
- **Group III (Negative control group)**: the children were allocated to switched-off Meridian Pen (*N* = 21).

### Intervention

Intervention appointments were limited to the hours between 10 am and 2 pm to minimize the effects of the diurnal variation of sAA [26]. All procedures were performed by a single trained and calibrated operator after undergoing a training period according to the criteria adopted. Training and calibration on the scales used were done and the intraclass correlation coefficient (ICC) score was 0.894.

**Fig. 1:**
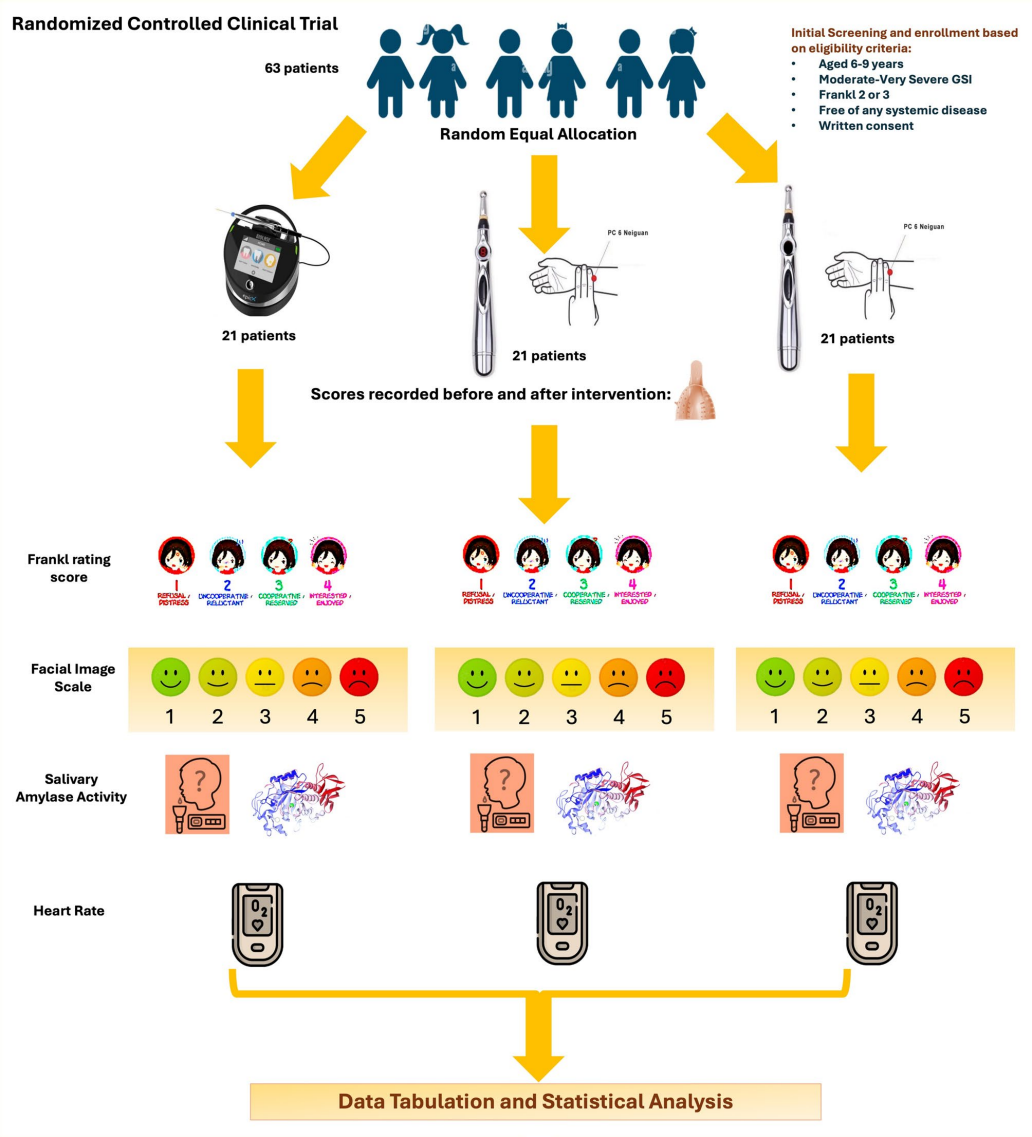
Randomized controlled clinical trial CONSORT flowchart

In the laser acupuncture group, protective eyewear was provided for the dentist, assistant, parent, and child. The Biolase (EpicX) diode laser, (California, USA), operates at 940 nm and delivers 4 J of energy (continuous mode). It is applied using a photobiomodulation handpiece (8 mm tip), positioned 3–4 mm from the PC6 acupoint for 40 s, with an output power of 100 mW to ensure a pain-free experience. The power density was 198.9 mW/cm², and the energy density (irradiance) was 7.95 J/cm². In the microcurrent electrical stimulation group, a meridian acupuncture pen (Meridian Acupuncture Therapy Electronic Pen, Ma Kuang, China) powered by an AA battery was used on the PC6 point for one minute [14], with fresh batteries for each patient to ensure consistent power of 3.7 V 300 mA±/50 mA. The negative control group received a deactivated meridian acupuncture pen applied without pressure. Right after the gag reflex control was applied in all three groups, an alginate dental impression of the upper arch was made (Fig. 1).

### Outcomes assessment

Dental anxiety was assessed both objectively and subjectively. Objectively, it was measured through changes in salivary sAA, heart rate (HR), and blood oxygen saturation (SaO2) using the pulse oximeter (PO 80, Beurer GmbH, Ulm, Germany). Subjectively, it was evaluated using the gag preventive index score (GPI) (Supplementary Table 2) [1, 5] the Frankl behavior rating scale (scores 1–4) [25]and the Facial Image Scale (FIS) [27], where the child indicated which face best represented their feelings at that moment—1 for very happy, 2 for happy, 3 for neutral, 4 for sad, and 5 for very sad. These assessments were conducted before the GR control method and after the upper arch impression procedure across all three groups.

### Salivary alpha-amylase sample collection (Fig. 2) ([28]–[29])

#### Before sample collection

Patients were asked to avoid high-sugar or acidic foods for at least one hour prior to sample collection to prevent alterations in saliva pH and bacterial growth. Strict timing for collecting sAA samples at the same time daily was enforced. Additionally, patients rinsed their mouths with water to eliminate food residues and waited at least 10 min post-rinsing to prevent sample dilution.

**Fig. 2:**
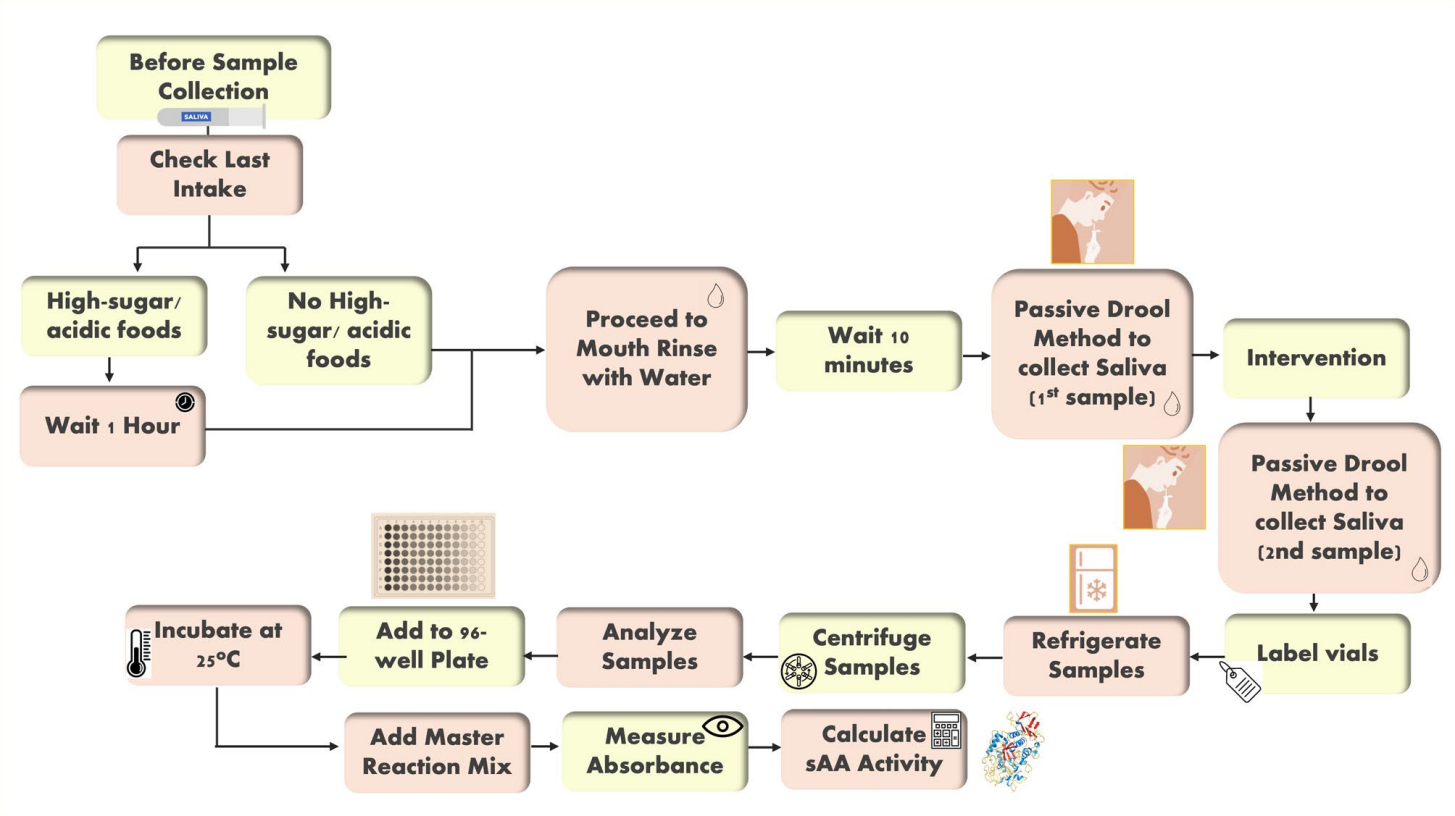
Salivary Alpha-Amylase Sample Collection Process

#### Saliva collection

Whole saliva was collected using the unstimulated passive drool method, considered the gold standard for biological testing as it yields the purest samples. Each patient provided two samples: one before the GR control method and another 7–10 min after the dental impression. Patients tilted their heads forward to allow saliva to pool in the mouth, which was then collected in a polypropylene vial, with a maximum of 2 mL per vial. Each vial was labeled with the patient’s serial number.

#### After sample collection

Samples were refrigerated within 30 min and centrifuged within four hours at 2000xg and 4 °C for 10 min, then stored at or below − 20 °C. The samples were analyzed at the Biochemistry Department Laboratory using an Alpha Amylase Enzymatic Assay (MAK009, Sigma-Aldrich, Germany) and a salivary amylase kit (Sali-Tubes, DRG instruments, GmbH, Germany). Alpha-amylase activity (nmol/min/ml) was determined via the enzyme kinetic method per the manufacturer’s instructions. Five microliters of each sample were added to a 96-well plate, mixed with Amylase Assay Buffer to reach a total volume of 50 µl. Five microliters of Amylase Positive Control Solution were also added and adjusted to the same volume, followed by incubation at 25 °C. A 100 µl Master Reaction Mix was added to each well and mixed thoroughly. Salivary alpha-amylase (sAA) activity was detected through the enzymatic hydrolysis of the substrate 2-chloro-4-nitrophenyl α-D maltotrioside. Absorbance at 405 nm was measured after 2–3 min for initial values (T-initial), with subsequent measurements every 5 min until the absorbance approached the highest standard (20 nm/well). sAA enzyme activity was calculated based on the increase in absorbance over time, using the formula:

(Δ sample) * sample dilution factor / (reaction time) * (volume), where Δ = absorbance test group T-final– absorbance test group T-initial. The positive control was p-nitrophenol, with activity determined using a standard curve.

### Statistical analysis

Descriptive statistics included means, standard deviations (SD), medians, interquartile ranges (IQR), frequencies, and percentages. Normality for quantitative variables was assessed using descriptive statistics, Q-Q plots, histograms, and Kolmogorov-Smirnov tests. Comparisons between the three study groups were conducted using one-way ANOVA or Kruskal-Wallis tests based on normality. Significant results were followed by multiple pairwise comparisons with Bonferroni correction. Within-group comparisons before and after intervention utilized paired t-tests or Wilcoxon signed-rank tests. Spearman correlation assessed differences in sAA and other variables, with significance set at *p* < 0.05. Data were analyzed using IBM SPSS (Version 26.0).

## Results

A total of 133 patients were screened for eligibility, and 63 participants met the inclusion criteria and were enrolled in our study, with 21 individuals in each group. Forty-five patients were excluded for being Frankl 1 or 4, ten for being special needs children, and fifteen because they did not require a maxillary-arch impression. The mean age of the participants was 7.17 years (± 0.90), and 61.9% were female. Additionally, 74.6% of the participants had a GSI score of 3, with no significant differences observed among the three groups.

Table 1 presents the comparison of sAA levels among the three study groups. Initially, there were no significant differences in sAA levels before the intervention (*p* = 0.15). However, after the intervention, the Meridian Pen group exhibited a significant reduction in mean levels (8.58 ± 1.67) compared to the Laser (13.10 ± 6.83) and Control (13.02 ± 5.76) groups, with a p-value of 0.009. The differences in sAA levels before and after the intervention were also significantly different among the groups (*p* = 0.001), with the Meridian Pen group showing a substantial decrease of -2.94 (± 1.65), while the Laser and Control groups had minor increases (0.38 and 0.11, respectively).

**Table 1:** Comparison of salivary alpha-amylase levels between the three study groups

	Meridian pen (*n* = 21)	Laser (*n* = 21)	Control (*n* = 21)	*P* value 1
	Mean (SD)			
**Before**	11.51 (1.42)	12.72 (2.76)	12.91 (2.92)	0.15
**After**	8.58 (1.67) **a**	13.10 (6.83) **b**	13.02 (5.76) **b**	0.009*
**Difference** ¥	-2.94 (1.65) **a**	0.38 (6.58) **b**	0.11 (4.25) **b**	0.001*
*P * **value 2**	0.001*	0.80	0.91	

P value 1: One-way ANOVA was used for comparing the three groups

*P* value 2: Paired t-test was used for comparing scores before and after the intervention within each group

¥: Kruskal Wallis test was used for comparing the difference between the three study groups

*statistically significant at p-value < 0.05

a, b: different letters denote significant differences between groups using Bonferroni correction

A comparison of Frankl scores among the three groups is presented in Table 2. Prior to the intervention, there were no significant differences in scores (*p* = 0.81), with most participants scoring a 3 (90.5% for Meridian Pen, 95.2% for Laser, and 90.5% for Control). Post-intervention results showed significant changes (*p* = 0.002), particularly in the Meridian Pen group, where 33.3% scored a 4, compared to 4.8% in the Laser group and none in the Control group. The mean scores after the intervention were highest in the Meridian Pen group (3.33 ± 0.48), followed by Laser (3.05 ± 0.22) and Control (2.95 ± 0.22). Significant improvement in Frankl scores was noted for the Meridian Pen group (*p* = 0.003).

**Table 2:** Comparison of *Frankl*, GSI/GPI, FIS and change in heart rate scores between the study groups

			Meridian pen (*n* = 21)	Laser (*n* = 21)	Control (*n* = 21)	*P* value 1
			*N* (%)			
**Frankl scores**	**Before**	**2**	2 (9.5%)	3 (14.3%)	1 (4.8%)	0.58
		**3**	19 (90.5%)	18 (85.7%)	20 (95.2%)	
		**4**	0 (0%)	0 (0%)	0 (0%)	
		**Mean (SD)**	2.90 (0.30)	2.86 (0.36)	2.95 (0.22)	
		**Median (IQR)**	3.00 (0.00)	3.00 (0.00)	3.00 (0.00)	
	**After**	**2**	0 (%)	0 (%)	1 (4.8%)	0.002*
		**3**	14 (66.7%)	20 (95.2%)	20 (95.2%)	
		**4**	7 (33.3%)	1 (4.8%)	0 (0%)	
		**Mean (SD)**	3.33 (0.48)	3.05 (0.22)	2.95 (0.22)	
		**Median (IQR)**	3.00 (1.00) **a**	3.00 (0.00) **b**	3.00 (0.00) **b**	
	*P * **value 2**		0.003*	0.10	1.00	
**GSI/GPI scores**	**Before (GSI)**	**Mean (SD)**	3.33 (0.48)	3.24 (0.44)	3.19 (0.40)	0.56
		**Median (IQR)**	3.0 (1.0)	3.0 (0.5)	3.0 (0.0)	
	**After (GPI)**	**Mean (SD)**	1.48 (0.60)	1.71 (0.46)	2.71 (0.56)	< 0.001*
		**Median (IQR)**	1.0 (1.0) **a**	2.0 (1.0) **a**	3.0 (1.0) **b**	
**FIS scores**	**Before**	**Mean (SD)**	3.57 (0.68)	3.90 (0.70)	3.57 (0.60)	0.14
		**Median (IQR)**	4.0 (3.0, 4.0)	4.0 (4.0, 4.0)	4.0 (3.0, 4.0)	
	**After**	**Mean (SD)**	2.05 (0.67)	2.29 (0.46)	3.14 (0.57)	< 0.001*
		**Median (IQR)**	2.0 (0.0) **a**	2.0 (1.0) **a**	3.0 (0.5) **b**	
	*P * **value 2**		< 0.001*	< 0.001*	0.003*	
**Heart rate percent change**	**Mean (SD)**		**-12.60 (4.50) a**	**-11.48 (2.19) a**	**-6.22 (2.75) b**	< 0.001*

*P* value 1: Kruskal Wallis test was used for comparing the three groups

*P* value 2: Wilcoxon signed ranks test was used for comparing scores before and after the intervention within each group

*statistically significant at p-value < 0.05

a, b: different letters denote significant differences between groups using Bonferroni correctiona

Table 2 also compares GSI/GPI, FSI scores and heart rate among the three groups. Before the interventions, the mean GSI scores were similar across groups (*p* = 0.56). However, after the intervention, significant differences emerged, with the Meridian Pen group showing the lowest mean GPI score (1.48), indicating better outcomes compared to the Laser (1.71) and Control (2.71) groups (*p* < 0.001). For FSI scores, no significant differences were noted before the intervention (*p* = 0.14), but after the intervention, the Meridian Pen (2.05) and Laser (2.29) groups scored significantly lower than the Control group (3.14), highlighting improved behavior (*p* < 0.001). The change in heart rate from pre- to post-intervention was significantly different among the groups (*p* < 0.001), with both the Meridian pen and laser groups showing similar percent reductions in heart rate (-12.60 and − 11.48, respectively), which were significantly greater than the control group’s percent reduction of -6.22 (*p* < 0.001).

Spearman correlation coefficients (Rho) between sAA level differences and various factors across three study groups are shown in Table 3. The Meridian Pen group shows no statistically significant correlations due to high p-values. However, the laser group has a significant negative correlation with the FIS (Rho = -0.62, *p* = 0.003). In the control group, heart rate shows a strong positive correlation (Rho = 0.85, *p* < 0.001).

**Table 3:** Spearman correlation between differences in salivary alpha-amylase levels and different factors in the three study groups

Difference (After– before)	Meridian pen	Laser	Control
	Rho (*p* value)		
**Frankl**	**0.15 (0.51)**	**-0.11 (0.64)**	**0.00001 (0.99)**
**GPI**	**-0.02 (0.94)**	**0.10 (0.68)**	**-0.24 (0.30)**
**FIS**	**0.03 (0.89)**	-0.62 (0.003*)	**0.05 (0.84)**
**Heart rate percent change**	**-0.01 (0.95)**	**0.33 (0.15)**	0.82 (< 0.001*)

Rho: Spearman correlation coefficient

*statistically significant at *p*-value < 0.05

## Discussion

The present study aimed to compare the efficacy of low-power laser acupuncture and microcurrent electrical stimulation in managing anxiety in children with excessive gag reflex (GR) during dental impressions. Our findings contribute to the literature on patient compliance in pediatric dentistry, particularly regarding anxiety and gagging during procedures.

Gagging is a common and distressing response in children during dental procedures. Previous research indicates that the interplay between stress, anxiety, and gagging behaviors necessitates effective management strategies for positive dental experiences and long-term oral health compliance [1–3, 7]. 

The efficacy of laser acupuncture lies in its ability to stimulate cellular processes through photobiomodulation, potentially increasing serotonin levels to help regulate anxiety and gag reflex responses [11, 14]. Microcurrent electrical stimulation operates similarly, influencing cellular activity and promoting relaxation at the acupoint [2, 3, 12]. Both methods are non-invasive and can enhance patient compliance and overall dental experiences [11, 30]. However, the acupuncture meridian pen is noted for its ease of use, while laser services tend to be more expensive [31]. 

Our results indicate that microcurrent electrical stimulation significantly reduces salivary alpha-amylase (sAA) levels, a stress biomarker [18–21]. Before intervention, sAA levels were similar across groups, but post-intervention, the microcurrent group showed a marked decrease, while the laser acupuncture and control groups had slight increases. This suggests that microcurrent stimulation may effectively alleviate stress responses. The reduction in sAA could correlate with improved patient compliance during dental impressions, supporting the idea that microcurrent electrical stimulation modulates physiological stress responses, consistent with findings by Alekhya et al. (2023) [30]. 

Our study demonstrates that microcurrent electrical stimulation effectively improves children’s behavior during dental procedures, evidenced by increased Frankl scores. Initially, differences among groups were minimal, but the Meridian Pen group showed the greatest improvement in cooperation. Both Meridian Pen and laser acupuncture interventions successfully reduced anxiety, as indicated by GPI, FSI, and heart rate scores, with the Meridian Pen achieving the lowest GPI score. Overall, these findings suggest that both methods reduce children’s anxiety during dental procedures, with the Meridian Pen being the most effective. This offers dental practitioners flexible options for managing gag reflexes in children based on individual needs and clinical contexts.

Regarding correlations, microcurrent electrical stimulation did not show statistically significant relationships between sAA levels and other variables, indicating a distinct effect on stress reduction. The significant change in salivary alpha-amylase (sAA) levels following the meridian pen intervention indicates its direct effect on stress or anxiety, as sAA serves as a biomarker for sympathetic nervous system activity; however, the lack of statistically significant correlations with other measured factors suggests that this effect may be independent of those variables, highlighting a complex relationship between physiological stress markers like sAA and psychological outcomes. In contrast, laser acupuncture exhibited a significant negative correlation between sAA levels and feelings of discomfort or anxiety, suggesting that the laser acupuncture treatment was comfortable for the children, resulting in less perceived discomfort or anxiety, despite the increase in sAA levels. This change acknowledges the complexity of the relationship between physiological markers and the children’s subjective experiences of comfort and anxiety. The control group revealed a strong positive correlation between sAA and heart rate, indicating a link between physiological stress responses and sAA levels.

While our study provides valuable insights, limitations include a small sample size, which may limit generalizability. Future studies should consider larger cohorts and longer follow-up periods to assess sustained effects. Additionally, exploring psychological factors influencing gagging, such as previous dental experiences, could enhance intervention tailoring. Qualitative assessments could further enrich our understanding of children’s perceptions of these interventions and their overall dental experiences.

On the other hand, having a range for saliva collection time (7–10 min) after the dental impression is regarded as essential and a strength of our study. This approach accommodates individual variability in saliva production, allows for stabilization of the oral environment, and ensures consistency in the collection process, ultimately enhancing the accuracy and validity of the study’s results [28, 29]. 

## Conclusion

This randomized controlled trial concludes that both low-power laser acupuncture and microcurrent electrical stimulation effectively reduce salivary alpha-amylase levels in children with excessive gag reflexes during dental impressions, indicating decreased dental anxiety. While both interventions improved behavioral scores, the Meridian Pen demonstrated the greatest reduction in alpha-amylase levels and the highest Frankl scores, indicating superior efficacy. These findings underscore the importance of non-invasive, patient-centered techniques in dental practice to improve compliance, reduce anxiety, and enhance care quality in pediatric dentistry.

## Electronic supplementary material

Below is the link to the electronic supplementary material.

Supplementary Material 1

Supplementary Material 2

Supplementary Material 3

## Data Availability

Data is provided within the manuscript or supplementary information files.

## Abbreviations

GR: Gag Reflex
PC6: Pericardium 6
HPA: Hypothalamic-Pituitary-Adrenal
sAA: Salivary α-amylase
