# Supplementary figures and images for: Comparative efficacy of laser and electroacupuncture on anxiety management and salivary alpha-amylase levels in pediatric dental patients with excessive gag reflexes: a randomized clinical trial
Source: BMC Oral Health. 2025 Jul 26;25:1254. doi: 10.1186/s12903-025-06630-x (PMC12297650; doi:10.1186/s12903-025-06630-x)

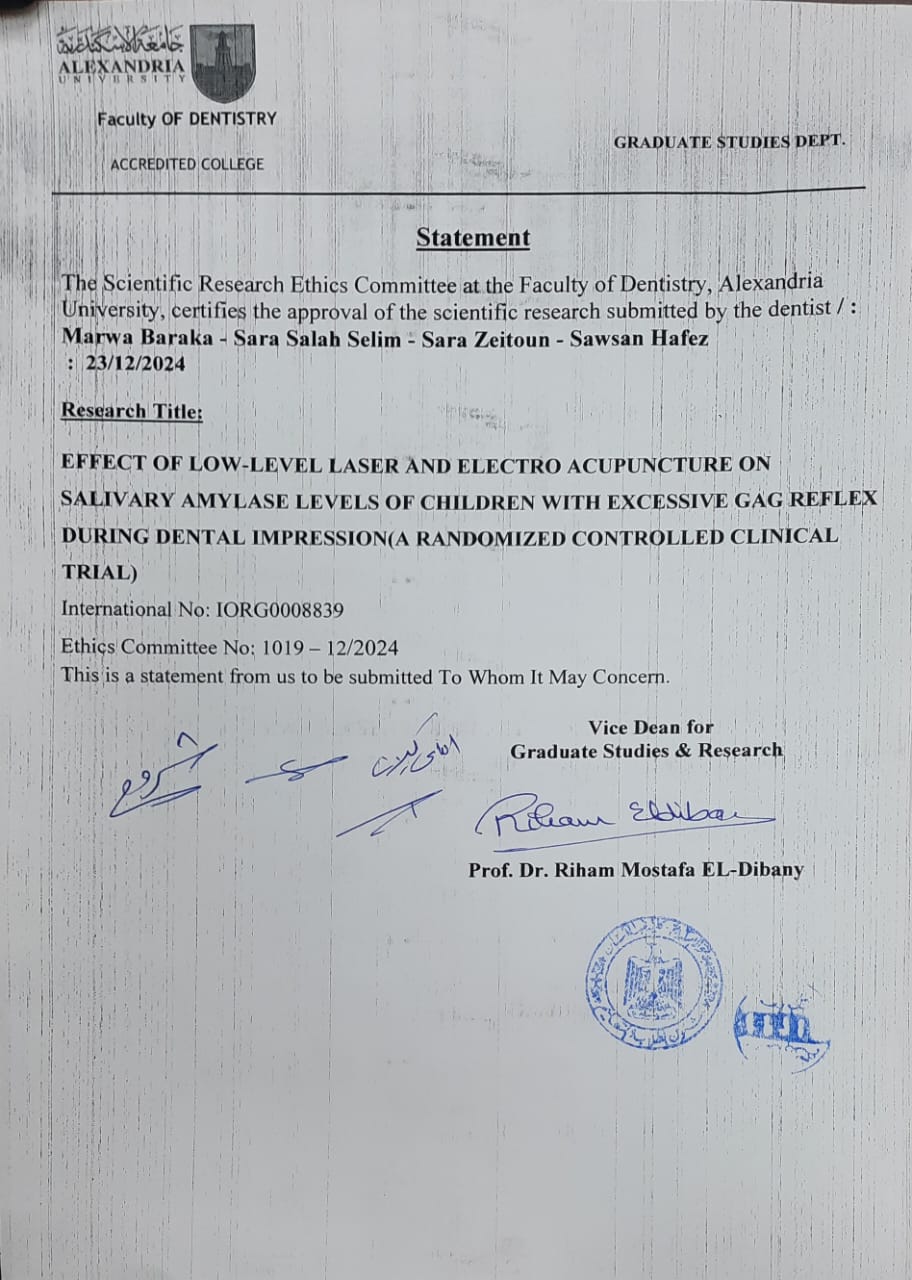

Supplement: Supplementary file 1 — Supplementary Material 1 [file 12903_2025_6630_MOESM1_ESM.jpeg]
