# Supplementary material for: Comparative efficacy of laser and electroacupuncture on anxiety management and salivary alpha-amylase levels in pediatric dental patients with excessive gag reflexes: a randomized clinical trial
Source: BMC Oral Health. 2025 Jul 26;25:1254. doi: 10.1186/s12903-025-06630-x (PMC12297650; doi:10.1186/s12903-025-06630-x)
Supplement: Supplementary file 3 — Supplementary Material 3 [file 12903_2025_6630_MOESM3_ESM.docx]

**Comparative Efficacy of Laser and Electroacupuncture on Anxiety Management and Salivary Alpha-Amylase Levels in Pediatric Dental Patients with Excessive Gag Reflexes: A Randomized Clinical Trial**

**Supplementary Table 1: Gag Severity Index Scoring**

| **The gagging reflex** |  |
| --- | --- |
| **I Very mild** | Occasional, and controlled by the patient |
| **II Mild** | Control is required by the patient with reassurance from the dental team |
| **III Moderate** | Consistent, and limits treatment options |
| **IV Severe** | Treatment is limited |
| **V Very severe** | Affecting patient behavior & dental attendance and making treatment impossible |

**Supplementary Table 2: Gag Preventive Index Scoring**

**
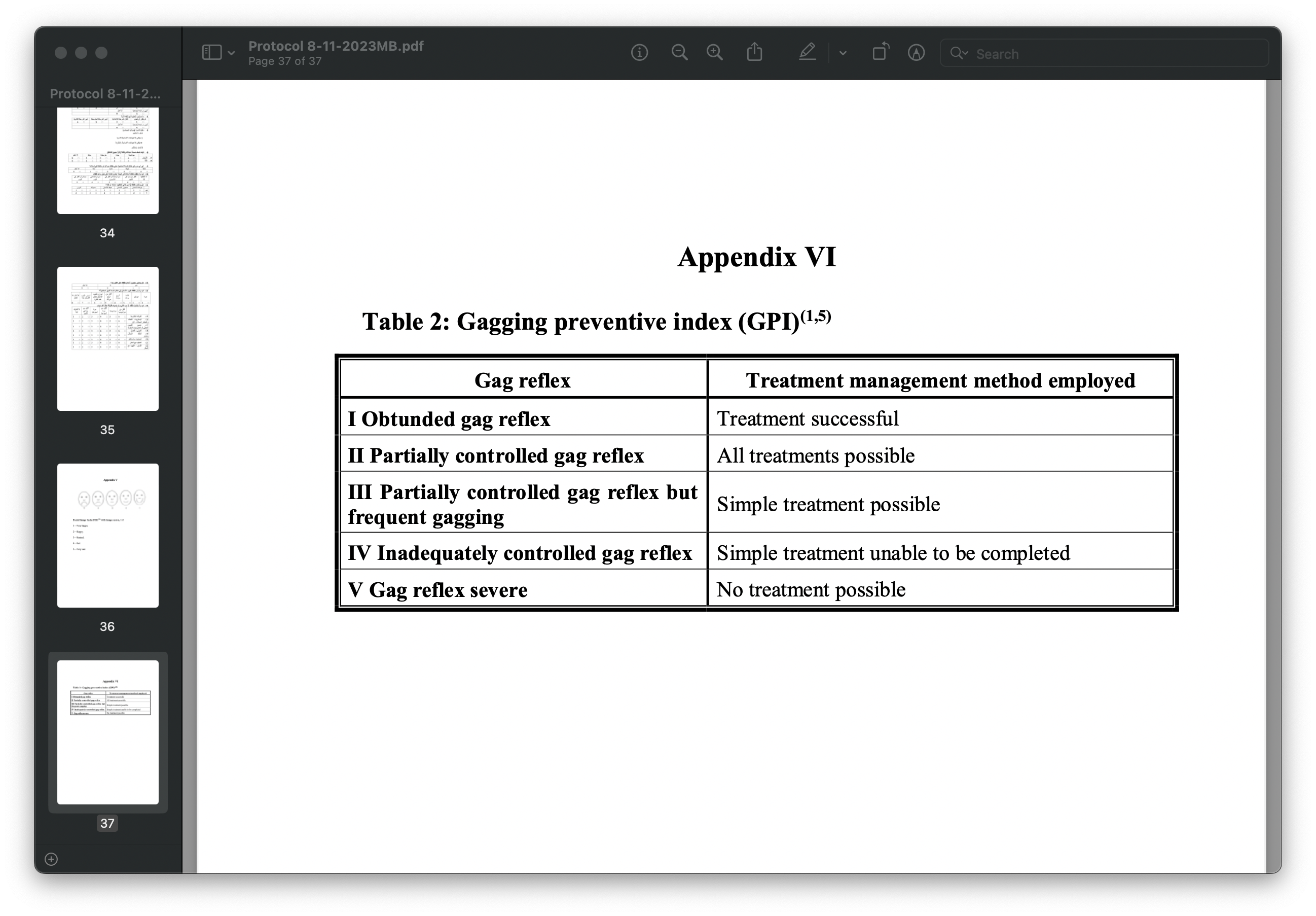
**
